# Sequence conservation of linker histones between chicken and mammalian species

**DOI:** 10.1016/j.dib.2014.10.002

**Published:** 2014-10-15

**Authors:** Bettina Sarg, Rita Lopez, Herbert Lindner, Inma Ponte, Pedro Suau, Alicia Roque

**Affiliations:** a Departamento de Bioquímica y Biología Molecular, Facultad de Biociencias, Universidad Autónoma de Barcelona, Bellaterra, Barcelona, Spain; b Division of Clinical Biochemistry, Biocenter, Innsbruck Medical University, Innsbruck, Austria

## Abstract

The percent identity matrices of two sequence multiple alignments between linker histones from chicken and mammalian species are described. Linker histone protein sequences for chicken, mouse, rat and humans, available on public databases were used. This information is related to the research article entitled “Identification of novel post-translational modifications in linker histones from chicken erythrocytes”published in the Journal of Proteomics [1].

## Specifications table

**Table:** 

Subject area	Biology
More specific subject area	Molecular evolution
Type of data	Tables
How data was acquired	Multiple sequence alignment using ClustalW running under MEGA 5.2
Data format	Analyzed
Experimental factors	Two multiple sequence alignments were performed corresponding to the H1.1–H1.5 clade and the H1.0/H5 clade [2].
	Initial methionine was removed from the protein sequence prior to the multiple sequence alignment.
Experimental features	Percent identity matrix contains the identity score from all of the pairwise comparisons calculated as the number of identities between two sequences divided by the length of the alignment and represented as a percentage.
Data source location	Not applicable
Data accessibility	All the sequences are registered at Uniprot or NCBI protein databases. The specific accession numbers are described below in Section 3.

**Table:** **Value of the data**.

**Fig. 1:**
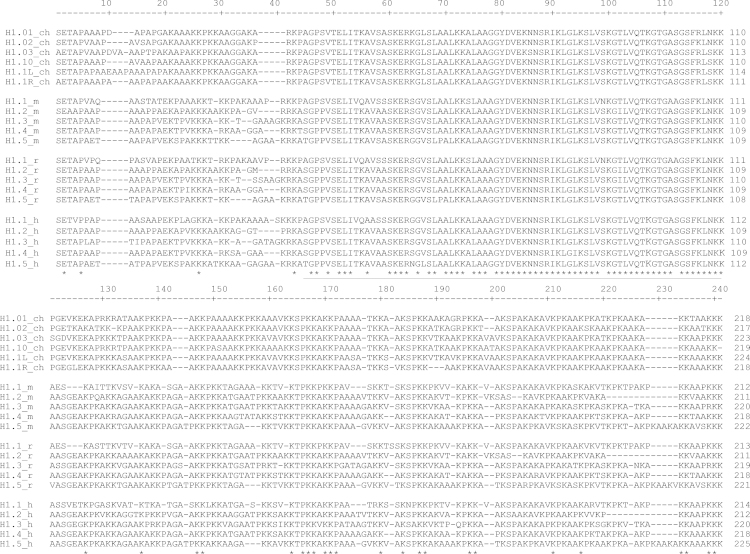
Multiple sequence alignment of chicken (ch) H1 subtypes and mammalian subtypes (H1.1–H1.5) from mouse (m), rat (r) and humans (h). The globular domain is underlined. Conserved aminoacids are denoted with an asterisk (^⁎^).

**Fig. 2:**
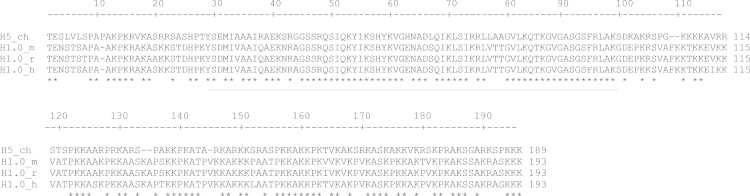
Multiple sequence alignment of chicken (ch) H5 and mammalian H1.0 from mouse (m), rat (r) and humans (h). The globular domain is underlined. Conserved aminoacids are denoted with an asterisk (^⁎^).

• Paralogous comparisons show that the six H1 subtypes from chicken (H1.01–H1.03, H1.10, H1.1L and H1.1R) are significantly closer, with an average percent identity of 90%, than mammalian paralogs (H1.1–H1.5) in any of the analyzed species, with an average percent identity of 73% (Table 1). The larger divergence of the mammalian paralogs in comparison with chicken paralogs suggests that the mammalian subtypes have acquired specific functions [3–5]. • Orthologous comparisons show that the H1 subtypes (H1.0–H1.5) from mouse, rat and humans, are highly conserved in interspecies comparison, reinforcing the idea of the functional differentiation of the H1 subtypes (Tables 1 and 2). The most divergent subtype is H1.1 with percentages of identity over 79%, while the more conserved subtypes are H1.0 and H1.4 with percentages of identity over 94% [3,4]. • All six chicken H1 subtypes have higher percentages of identity when compared with H1.4 (more than 65%), suggesting that its function is conserved between avian and mammalian species (Table 1). • The percentage of identity between H5, an avian specific linker histone associated with terminal differentiation and its mammalian counterpart H1.0 is over 66%, indicating function conservation (Table 2). • Figs. 1 and 2 show that the globular domain is the most conserved region of the linker histones [3,4].

## 1 Experimental design, materials and methods [1]

Two separate sequence alignments were performed with chicken linker histones (see Figs. 1 and 2). The first included chicken H5 and H1.0 from mouse, rat and humans (Fig. 1), and the second included the six chicken H1 subtypes and H1 subtypes (H1.1–H1.5) from the above specified species (Fig. 2). In both cases an initial alignment was obtained using ClustalW running under MEGA 5.2. The alignment was then optimized by visual inspection. The pairwise identity score was calculated by the number of identities between two sequences divided by the length of the alignment and represented as a percentage. The accession numbers for the chicken (ch), mouse (m), rat (r) and human (h) sequences were as follows: H5_ch, Uniprot: P02259; H1.01_ch, Uniprot: P08284; H1.02_ch, Uniprot: P09987; H1.03_ch, Uniprot: P08285; H1.10_ch, Uniprot: P08286; H1.1L, Uniprot: P08287; H1.1R, Uniprot: P08288; H1.0_m, Uniprot: P10922; H1.1_m, Uniprot: P43275; H1.2_m, Uniprot: P15864; H1.3_m, Uniprot:P43277; H1.4_m, Uniprot: P43274; H1.5_m, Uniprot: P43276; H1.0_r; Uniprot: P43278; H1.1_r, NCBI Protein: NP_001099583; H1.2_r, NCBI Protein:XP_001071565; H1.3_r, NCBI Protein:XP_001072089; H1.4_r, NCBI Protein: x67320, H1b_r, NCBI Protein: NP_001102887; H1.0_h, Uniprot: P07305; H1.1_h, Uniprot: Q02539; H1.2_h, Uniprot: P16403; H1.3_h, Uniprot: P16402; H1.4_h, Uniprot:P10412; H1.5_h, Uniprot: P16401.

## Figures and Tables

**Table 1:** Percent identity matrix of the avian and mammalian H1 subtypes.

H1.01_ch	100.00									
H1.02_ch	91.66	100.00								
H1.03_ch	88.33	86.25	100.00							
H1.10_ch	92.50	90.83	89.16	100.00						
H1.1L_ch	89.16	86.66	89.58	90.00	100.00					
H1.1R_ch	90.00	87.08	89.58	90.41	92.08	100.00				
H1.1_m	60.41	61.66	60.00	59.58	60.41	61.25	100.00			
H1.2_m	62.50	62.91	60.83	63.74	62.08	62.91	63.33	100.00		
H1.3_m	66.66	66.25	64.58	65.83	64.16	66.25	68.75	78.75	100.00	
H1.4_m	70.00	67.91	65.41	68.33	65.00	67.50	68.75	78.75	87.08	100.00
H1.5_m	65.00	64.16	62.91	64.16	61.66	63.74	67.91	69.16	81.25	77.50
H1.1_r	60.41	60.83	58.75	58.75	60.41	60.00	95.00	62.08	66.25	67.91
H1.2_r	62.50	62.91	60.83	63.74	62.08	62.91	64.16	97.91	78.75	79.58
H1.3_r	65.00	65.00	63.33	64.16	62.91	65.00	66.25	75.83	94.58	83.33
H1.4_r	69.16	67.50	65.41	67.50	65.00	67.50	68.75	80.41	88.33	97.08
H1.5_r	63.74	62.50	61.66	62.50	60.00	62.50	66.25	67.08	80.00	76.25
H1.1_h	60.41	60.83	59.16	59.58	60.41	60.83	80.83	64.58	70.83	70.00
H1.2_h	64.58	65.00	62.50	65.00	62.91	63.74	63.74	87.91	80.83	80.00
H1.03_h	65.41	65.00	63.33	64.16	62.08	64.16	66.66	75.00	88.75	84.16
H1.4_h	70.00	69.16	67.08	70.00	67.08	69.58	69.58	81.25	90.00	94.16
H1.5_h	67.50	67.08	64.58	67.08	65.00	67.08	64.58	72.50	81.25	81.66
	H1.01_ch	H1.02_ch	H1.03_ch	H1.10_ch	H1.1L_ch	H1.1R_ch	H1.1_m	H1.2_m	H1.3_m	H1.4_m


H1.01_ch											
H1.02_ch											
H1.03_ch											
H1.10_ch											
H1.1L_ch											
H1.1R_ch											
H1.1_m											
H1.2_m											
H1.3_m											
H1.4_m											
H1.5_m	100.00										
H1.1_r	66.25	100.00									
H1.2_r	69.16	62.91	100.00								
H1.3_r	77.91	64.58	75.83	100.00							
H1.4_r	77.91	67.91	81.25	84.58	100.00						
H1.5_r	96.25	65.00	67.08	76.66	76.66	100.00					
H1.1_h	68.75	79.16	65.00	69.16	70.83	67.50	100.00				
H1.2_h	71.25	62.50	87.91	77.50	80.41	69.16	65.00	100.00			
H1.03_h	75.83	65.41	75.00	84.16	84.58	74.58	69.16	78.33	100.00		
H1.4_h	80.00	68.75	81.25	85.83	95.41	78.75	72.50	81.66	85.83	100.00	
H1.5_h	91.25	63.74	72.50	77.91	82.08	89.16	68.33	72.91	77.08	84.16	100.00
	H1.5_m	H1.1_r	H1.2_r	H1.3_r	H1.4_r	H1.5_r	H1.1_h	H1.2_h	H1.3_h	H1.4_h	H1.5_h

The pairwise identity score was calculated by the number of identites between two sequences, divided by the length of the alignment represented as a percentage. Abbreviations: ch, chicken; m, mouse; r, rat and h, humans.

**Table 2:** Percent identity matrix of avian H5 and mammalian H1.0.

H5_ch	100.00			
H1.0_m	67.01	100.00		
H1.0_r	66.49	97.93	100.00	
H1.0_h	67.01	94.84	94.32	100.00
	H5_ch	H1.0_m	H1.0_r	H1.0_h

The pairwise identity score was calculated by the number of identites between two sequences, divided by the length of the alignment represented as a percentage. Abbreviations: ch, chicken; m, mouse; r, rat and h, humans.
